# Oral Anticoagulants in Chronic Thromboembolic Pulmonary Hypertension: Tradition or Innovation?

**DOI:** 10.3390/jcdd12070271

**Published:** 2025-07-16

**Authors:** Domenico Laviola, Giovanna Manzi, Tommaso Recchioni, Maria Cristina Luise, Valentina Mercurio, Alexandra Mihai, Roberto Badagliacca, Silvia Papa, Carmine Dario Vizza

**Affiliations:** 1 Department of Clinical, Internal, Anesthesiology and Cardiovascular Sciences, Policlinico Universitario Umberto I, Sapienza University of Rome, 00161 Rome, Italy; domenico.laviola@uniroma1.it (D.L.); tommaso.recchioni@uniroma1.it (T.R.); alexandra.mihai@uniroma1.it (A.M.); roberto.badagliacca@uniroma1.it (R.B.); silvia.papa@uniroma1.it (S.P.); dario.vizza@uniroma1.it (C.D.V.); 2 Department of Translational Medical Sciences, Federico II University of Naples, 80138 Naples, Italy; mcluise06@gmail.com (M.C.L.); valentina.mercurio@unina.it (V.M.)

**Keywords:** chronic thromboembolic pulmonary hypertension, direct oral anticoagulants, DOACs

## Abstract

Chronic thromboembolic pulmonary hypertension (CTEPH) can complicate the clinical course of patients with acute pulmonary embolism, with a variable prevalence of 0.5–4%. Beyond specific therapeutic strategies, including pulmonary endarterectomy, balloon pulmonary angioplasty and pulmonary vasodilators, lifelong anticoagulation still represents the mainstay of treatment for this condition. The main historical experience supports the use of vitamin K antagonists (VKAs) in CTEPH patients; conversely, the efficacy and safety of direct oral anticoagulants (DOACs) in this setting are unclear. Growing experience, mainly from small studies and registries, is improving our knowledge, showing that DOACs may represent a valid and promising alternative to warfarin in CTEPH patients. Therefore, in the management of cases with a newly diagnosed CTEPH, clinicians are very often in the difficult position of (a) having to choose which anticoagulant to initiate in anticoagulant-naïve patients or (b) having to evaluate whether it is necessary to switch to a VKA in patients already on DOACs. This article aims to critically summarize the current evidence comparing DOACs and VKAs in CTEPH, discussing their efficacy and safety profiles and exploring their clinical applicability.

## 1. Introduction

Chronic thromboembolic pulmonary hypertension (CTEPH), the most severe long-term complication of acute pulmonary embolism, has an estimated incidence of 5–6 cases per million inhabitants per year. The increase in pulmonary vascular resistance (PVR) that characterizes this form of pulmonary hypertension (PH) results primarily from the presence of unresolved thromboembolic material in the pulmonary arteries and secondarily from subsequent microvasculopathy, which also involves the pulmonary capillaries and veins. The acronym AMEND, proposed during the last World Symposium on PH, expresses the steps towards recovery recommended for the management of CTEPH patients well: anticoagulation (A), multidisciplinary team review (M), pulmonary endarterectomy (E), non-surgical treatments (including medical therapy and balloon pulmonary angioplasty) (N), and regular follow-up, which clinicians should not forget after any intervention (D = do not forget) [1].

The description of all possible therapeutic strategies for the treatment of CTEPH is not the aim of this short communication, which is rather focused on the topic of anticoagulation.

## 2. Methods

A search was conducted across PubMed, Scopus, and Web of Science databases by at least two independent authors (T.R. and G.M) using combinations of the keywords “CTEPH”, “chronic thromboembolic pulmonary hypertension”, “DOAC”, “direct oral anticoagulants” and “VKA.” The search covered publications from January 2015 to March 2025. To achieve a search strategy with the maximum sensitivity, we combined all the previous terms either in “key word” or “MeSH terms” form, to reduce the risk of missing evidence. Moreover, the reference lists of all included articles, previous literature reviews on the topic, and top hits from Google Scholar were reviewed for the further identification of potentially relevant studies and were assessed using the inclusion and exclusion criteria. We included original articles and meta-analyses reporting on the efficacy or safety of DOACs versus VKAs in CTEPH (Table 1). Case reports, reviews without original data and studies on acute PE without separate CTEPH analysis were excluded.

## 3. Results and Discussion

**Figure 1:**
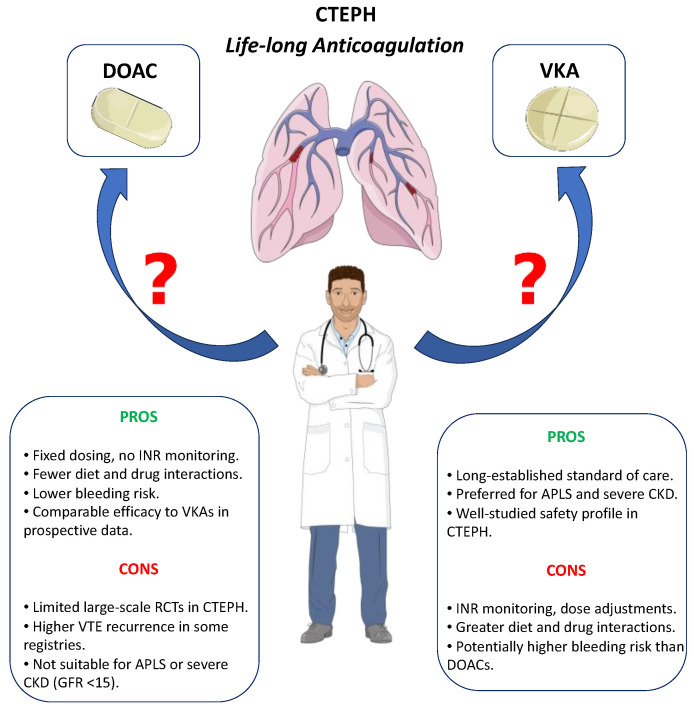
A comparison between direct oral anticoagulants (DOACs) and vitamin K antagonists (VKAs) in the management of chronic thromboembolic pulmonary hypertension (CTEPH): DOACs are a promising alternative with advantages such as fixed dosing, fewer interactions, and potentially lower bleeding risk; However, their use is limited by the lack of robust randomized trials in CTEPH, a higher VTE recurrence rate in some studies and contraindications in conditions like antiphospholipid syndrome (APLS) and severe renal impairment (CKD). VKAs remain the established standard of care, especially for high-risk subgroups, with a well-known safety profile. Their drawbacks include the need for frequent INR monitoring, dose adjustments, and higher susceptibility to dietary and drug interactions.

Lifelong therapeutic anticoagulation is strongly recommended in CTEPH due to the recurring nature of pulmonary thromboembolism (PE) and the challenges in clot resolution associated with this condition, but uncertainty still exists about the type of anticoagulant to be preferred (Figure 1).

Vitamin K antagonists (VKAs) are the first choice in this setting thanks to their long clinical use: they are proven to be effective in reducing blood clots and are considered the standard of care in special situations, such as antiphospholipid syndrome, which affects about 10% of CTEPH patients [1]. However, the drawbacks typically associated with VKAs (e.g., need for frequent blood samples for the international normalized ratio control, dose adjustments, a narrow therapeutic range, and diet or drug interactions) have prompted researchers to investigate the potential role of novel oral anticoagulants in CTEPH patients. DOACs are currently approved for stroke prevention in atrial fibrillation [11] and for the acute treatment and secondary prevention of venous thromboembolism (VTE), but they are not recommended as a first choice for CTEPH patients due to the paucity of randomized controlled trials (RCTs) specifically designed to test their safety and efficacy in this setting. Nevertheless, DOACs’ use in CTEPH is progressively increasing in clinical practice: the class IA recommendation for DOACs in the 2019 European Society of Cardiology (ESC) guidelines for the diagnosis and management of acute pulmonary embolism and the reluctance of many clinicians to switch from DOACs to VKAs in patients who develop CTEPH more than 3 months after the acute embolic event may partly explain this finding [12].

When choosing between VKAs and DOACs for CTEPH patients, PH specialists face two main challenges:

- are DOACs as effective as VKAs in preventing further venous thromboembolic events?
- do DOACs have the same safety profile as VKAs?

Answers can currently be gleaned mainly from case series, registries or meta-analyses, as data from randomized clinical trials are currently poor. Herein, we report the main experiences.

Gavilanes-Oleas et al. firstly analyzed a small cohort of 20 CTEPH patients, both operable and inoperable, receiving rivaroxaban (n = 16), dabigatran (n = 3) and apixaban (n = 1) [2]. During the follow-up period of 20 ± 14 months, no episode of venous thromboembolism (VTE) recurrence was reported; the only major bleeding resulting in the death of the patient occurred after a traumatic fall. Later, a retrospective study by Sena et al., including 501 CTEPH patients (8.2% classified as inoperable), showed that the rate of recurrent thromboembolism and non-relevant bleeding did not differ significantly between the DOACS and VKA groups over an observation period of 9.0 ± 8.5 years. Conversely, the rate of major bleedings, defined as a reduction in hemoglobin levels to >2.0 g/dL, bleedings affecting vital organs or bleedings requiring transfusion of >2 U of blood, was significantly higher in the warfarin group, likely due to unfavorable International Normalized Ratio (INR) control and higher HAS-BLED scores in this population.

In line with these results, the CTEPH AC registry [3], a large prospective observational cohort conducted in Japan, including 481 patients (52%) treated with DOACs and 446 patients (48%) receiving warfarin, showed the (a) comparable efficacy of DOACs and VKAs in the prevention of symptomatic VTE and (b) DOACs superiority in terms of safety due to the lower rate of major bleeding (*p* = 0.007).

Apparently conflicting data on VTE recurrence from other studies have raised some concern among PH specialists.

In 2019, Bunclark and colleagues published the results of a retrospective analysis that included consecutive CTEPH patients undergoing PEA, with 808 (75% of the overall population) treated with VKA and 204 (19%) treated with DOACs. While post-pulmonary endarterectomy outcomes (such as improvements in hemodynamics and functional status) and the incidence of bleeding events were unaffected by anticoagulation, CTEPH patients treated with DOACS experienced a significantly higher rate in VTE recurrence compared to VKAs (ten events corresponding to 4.64%/person year in the DOAC group vs. 12 events corresponding 0.76%/person year in the VKA group; *p* = 0.008) [4]. A more detailed analysis of the study shows that four VTE events in the DOAC arm occurred in the context of subtherapeutic dosing and that another event occurred in a patient with underlying thrombophilia (antiphospholipid syndrome), and that there were no VTE-related deaths in either group.

Higher rates of embolic and/or thrombotic events in DOAC users compared to VKA were reported also by Humbert and colleagues, who analyzed data from EXPERT, a non-interventional registry aimed at monitoring the long-term safety of riociguat in clinical practice in CTEPH patients. In a study including 198 CTEPH patients receiving a DOAC at baseline, mainly rivaroxaban, and 683 patients receiving a VKA, the authors found that hemorrhagic events appeared similar in the two groups, whereas the exposure-adjusted rate of embolic and thrombotic events was higher with DOACs than with VKAs [5]. The anticoagulant doses were not routinely recorded in this registry, so it cannot be assumed that the higher rate of embolic events was related to subtherapeutic doses of DOACs, as reported above. However, it is interesting to note the difference in the rate of anticoagulant discontinuation in the two groups, which may have affected the results (7.0% in the VKA groups vs. 15.2% in the DOAC group).

A retrospective study by Jeong et al. has further contributed to concerns regarding the use of DOACs in the prevention of recurrent thromboembolism in CTEPH [6]. The review of specimens from 405 consecutive CTEPH patients undergoing PEA showed that patients treated with DOACs were more likely to have a concomitant thrombus removed at the time of surgery compared to the non-DOAC treated group (13.3% vs. 6.7% respectively). Again, the authors could not evaluate the adequacy of each anticoagulation method due to the lack of information on the specific dosage of each DOAC or on the INR ranges.

In this confusing landscape, randomized trials and metanalyses helped clinicians shed light on the topic.

Barati et al. analyzed 96 CTEPH patients all receiving warfarin before endarterectomy. Twenty-four hours after PEA, 61 patients continued taking warfarin while the other 35 received rivaroxaban (2:1 allocation ratio). No significant differences between the groups were reported regarding thrombosis recurrence, bleedings, hospital re-admission and mortality in the first, third and sixth month after surgery [7].

The KABUKI, a multicenter, single-blind, randomized, warfarin-controlled trial undertaken in Japan, enrolled 74 CTEPH patients who had undergone PEA or balloon pulmonary angioplasty: 37 were randomized to edoxaban and 37 patients were assigned to warfarin [8]. The ratio of pulmonary vascular resistance (PVR) at week 48 to PVR at baseline was 0.93 (95% CI, 0.86–1.01) in the edoxaban group and 1.01 (95% CI, 0.93–1.10) in the warfarin group, thus demonstrating that edoxaban was non-inferior to warfarin in preventing worsening PVR in CTEPH patients (*p* < 0.0001 for non-inferiority). Also, the incidence of symptomatic venous thromboembolism and clinically relevant bleeding events was similar between the two groups.

Both randomized trials were included in two meta-analyses whose results supported the use of DOACs in CTEPH. The first one conducted by Salazar et al. included ten studies for a total of 3835 CTEPH patients and showed no statistically significant difference between anticoagulation strategies in terms of morbidity and mortality, recurrent venous thromboembolism and major bleeding [9]. The second metanalysis, recently published by Zhang and colleagues, focused the analysis on two RCTs and two prospective cohorts with a total of 2038 CTEPH patients, 751 treated with DOACs and 1287 receiving VKAs. The two groups experienced similar rates of effectiveness, including all-cause mortality and VTE recurrence. The rate of bleeding was slightly lower in patients taking DOACs, although the difference was not statistically significant [10].

The last consideration concerns the use of antiplatelet therapy in addition to anticoagulants in CTEPH patients. The rationale lies in the high activation of the platelet system found in this setting. This interesting finding was first demonstrated by Yaoita N. and colleagues, who found increased levels of P-selectin and PAC-1-positive platelets in patients with CTEPH compared to non-PH cases [13], and was later supported by Remkovà et al. [14], who demonstrated lower platelet counts, a higher mean platelet volume (MPV) and higher spontaneous platelet aggregation in CTEPH patients compared to healthy subjects. However, current data are insufficient to clarify the role of antiplatelet treatment in preventing the progression of CTEPH and further investigation in this setting is warranted.

## 4. Conclusions

While the use of non-vitamin K antagonist oral anticoagulants (DOACs) as first-line therapy is well established in atrial fibrillation and acute pulmonary embolism, their role in the management of chronic thromboembolic pulmonary hypertension (CTEPH) remains less defined. Available data from retrospective studies, registries, and a limited number of randomized controlled trials suggest that DOACs may represent a safe and effective alternative to vitamin K antagonists (VKAs) in selected CTEPH patients, offering advantages such as fixed dosing and a potentially lower risk of major bleeding.

However, several limitations must be acknowledged. Most studies are observational and heterogeneous in terms of patient population, disease severity, anticoagulant type and dose, and the definition of outcomes. Furthermore, suboptimal DOAC dosing, incomplete information on adherence, and a lack of data in high-risk subgroups (e.g., antiphospholipid syndrome, severe renal impairment) may affect the generalizability of results.

In conclusion, clinicians should adopt a personalized approach when choosing the type of anticoagulant in CTEPH, carefully balancing individual thrombotic and bleeding risk profiles and comorbidities. Further high-quality, randomized studies are warranted to definitively place DOACs in the therapeutic algorithm of CTEPH and to establish the role of antiplatelet therapy.

## Figures and Tables

**Table 1:** Studies on DOACs vs. VKA in CTEPH.

Author	Year of Publication	Study Design	Patients’ Characteristics	Type of Anticoagulation (Number of Patients)	Effectiveness in Prevention of VTE Recurrence *	Safety Profile *
Gavilanes-Oleas et al. [2]	2018	Retrospective	Non-operated	Rivaroxaban (16), Dabigatran (3), Apixaban (1)	No VTE recurrence	1 major bleeding after traumatic fall
Hosokawa et al. [3]	2023	Prospective	Mixed	DOACs (481), VKA (446)	Similar VTE recurrence	Fewer major bleedings with DOACs
Bunclark et al. [4]	2020	Retrospective	Operated	DOAC (204), VKA (808)	Higher VTE recurrence with DOACs	
Humbert et al. [5]	2022	Prospective registry	Non-operated	DOAC (198), VKA (683)	Higher embolic events with DOACs	Similar bleeding
Jeong et al. [6]	2022	Retrospective	Operated	Not specified	More residual thrombus in DOAC group during PEA	
Barati et al. [7]	2023	Prospective	Operated	Rivaroxaban (35), Warfarin (61)	No significant differences in outcomes	
KABUKI Trial [8]	2024	RCT	Operated	Edoxaban (37), Warfarin (37)	Similar VTE	Similar bleeding rates
Salazar et al. [9]	2024	Meta-analysis of data from 10 studies	Mixed	Mixed DOACs vs. VKA	Similar VTE	Similar bleeding rates
Zhang et al. [10]	2024	Meta-analysis	Mixed	DOAC (751), VKA (1287)	Similar effectiveness	Trend to lower bleeding with DOACs

* The table reports the main studies analyzing efficacy and safety of DOACs in patients with CTEPH. Green: in favor of DOAC; yellow: no differences between DOAC and VKA; red: in favor of VKA.

## Data Availability

No new data were created or analyzed in this study. Data sharing is not applicable to this article.

## Abbreviations

CTEPH	Chronic thromboembolic pulmonary hypertension
DOAC	Direct oral anticoagulants
ESC	European Society of Cardiology
INR	International Normalized Ratio
PH	Pulmonary hypertension
RCT	randomized controlled trials
VKA	Vitamin K antagonists
VTE	venous thromboembolism
